# Age-related changes in cellular electrophysiology and calcium handling for atrial fibrillation

**DOI:** 10.1111/jcmm.12084

**Published:** 2013-07-09

**Authors:** Guo-Jun Xu, Tian-Yi Gan, Bao-Peng Tang, Zu-Heng Chen, Tao Jiang, Jian-Guo Song, Xia Guo, Jin-xin Li

**Affiliations:** a Department of Cardiology First Affiliated Hospital, Xinjiang Medical UniversityUrumqi, China; b Department of Animal Experiment First Affiliated Hospital, Xinjiang Medical UniversityUrumqi, China; c Laboratory of Electrophysiology First Affiliated Hospital, Xinjiang Medical UniversityUrumqi, China; d Department of Molecular Biology, Xinjiang Medical UniversityUrumqi, China

**Keywords:** Atrial fibrillation, Ageing, L-type Ca^2+^ current, Cellular electrophysiology, Ca^2+^ handling proteins

## Abstract

This study was to investigate whether or not the dysfunction of atrial repolarization and abnormality of the intracellular Ca^2+^ handling protein was augmented with ageing. Four groups of dogs were studied, adult and aged dogs in sinus rhythm (SR) and atrial fibrillation (AF) induced by rapid atrial pacing. We used whole cell patch clamp recording techniques to measure L-type Ca^2+^ current in cardiomyocytes dispersed from the left atria. Expressions of the Ca^2+^ handling protein were measured by real-time quantitative reverse transcription-polymerase chain reaction and Western blot methods. Cardiomyocytes from old atria showed longer action potential (AP) duration to 90% repolarization, lower AP plateau potential and peak L-type Ca^2+^ current densities at both age groups in SR. AF led to a higher maximum diastolic potential, an increase of amplitude of phase 0, decreases of AP duration to 90% repolarization, plateau potential and peak L-type Ca^2+^ current densities. Compared to the adult group, mRNA and protein expressions of the L-type calcium channel a1c were decreased, whereas expressions of calcium adenosine triphosphatase were increased in the aged group. Compared to SR group, expressions of Ca^2+^ handling protein except for phospholamban were significantly decreased in both age groups with AF. We conclude that these ageing-induced electrophysiological and molecular changes showed that general pathophysiological adaptations might provide a substrate conducive to AF.

## Introduction

It is well-established that the incidence and prevalence of atrial fibrillation (AF) increases with age [1, 2]. Although the mechanism underlying AF has been investigated in humans and in animal models, cellular electrophysiological and molecular changes that render the atria of aged individuals more susceptible to AF than those of adults remain poorly understood.

Previous studies have reported that L-type Ca^2+^ current (I_Ca-L_), the major action potential (AP) plateau current, is reduced in aged canine right atrial cells compared with adults [3]. Recent evidence has shown that AP duration (APD) in right atrial cardiomyocytes is prolonged with age and the AP plateau becomes increasingly negative with age [4, 5]. In fact, AF mainly come from left atria, rather than right atria [1–3, 6], as we well know in clinical practice. However, there are no published data on the effects of age on left atrial I_Ca-L_. While it is clear that abnormal intracellular Ca^2+^ dynamics may underlie electrical remodelling in specialized aged atrial cells [6–9], as yet there has been no systematic study of intracellular Ca^2+^ handling in aged left atria. Some scholars have suggested that ageing reduces the activity of proteins influencing the calcium homeostasis in persistent human AF [10–14]. However, because of the lack of aged control group, it is less obvious whether the change is because of age, the presence of AF or other reasons. We suggest that dysfunction of atrial repolarization and abnormality of the intracellular Ca^2+^ handling protein (L-type calcium channel a1c (LVDCC_a1c_), calcium adenosine triphosphatase (Ca^2+^-ATPase), ryanodine receptor type-2 (R_Y_R_2_), inositol triphosphate receptor type-1(IP_3_R_1_) or ancillary proteins phospholamban (PLN) in the microenvironment increase with ageing, and thus create a substrate for initiation and maintenance of AF. The aim of the present study was to determine whether the general pathophysiological mechanisms of normal old atria provide a substrate conducive to atrial arrhythmias, particularly AF.

## Materials and methods

### Animal preparation

Fourteen adult (1–3 years) and 14 aged (more than 8 years) mongrels, weighing 18–26 kg each, were obtained from the Animal Center (Xinjiang Medical University, Urumqi, China). The ages of the dogs were estimated by a veterinarian based on standard measures for age, including dentition, coat, eyes and musculoskeletal and conformational descriptors. The dogs were kept in a temperature-controlled house under a 12 h light/dark cycle and fed a standard laboratory diet and water *ad libitum*. The Animal Care and Use Committee of the Xinjiang Medical University have approved all experiments in accordance with the Declaration of the National Institutes of Health Guide for Care and Use of Laboratory Animals (Publication No. 85-23, revised 1985).

Six-lead electrocardiogram (ECG) measurements were performed on conscious dogs resting quietly to confirm sinus rhythm (SR). Echocardiograms were performed to exclude structural heart disease. Dogs of each age were randomly divided into four groups of seven animals, the adult SR group and the aged SR group, the adult AF group and the aged AF group. AF was induced by chronic rapid atrial pacing and defined as persistence of AF for at least 5 days.

### Induction of AF

Animals were anaesthetized with pentobarbital sodium (30 mg/kg i.v.) and ventilated with isoflurane, 1.5–2%, and O_2_, 2 l/min. Morphine sulphate 0.15 mg/kg was injected into the epidural space to maintain post-operative analgesia. Using sterile techniques, a right intercostal thoracotomy was performed, the pericardium was opened and the heart was suspended in a pericardial cradle. A lead was attached to the epicardium of the left atrial appendage. The lead was tunnelled subcutaneously and connected to a Pulse Generator (Department of Electronic Engineering, Fudan University, Shanghai, China). Pulse generators were implanted in subcutaneous pockets on the left posterior chest wall. After the incisions were closed and the dogs recovered from anaesthesia, they were monitored for 2–3 days in the recovery room before being moved to routine care. The dogs were prophylactically treated with cefazolin, 25 mg/kg IV twice daily for 2 days after surgery. They were allowed to stabilize for 1 week and then were paced from the left atrial appendage at 600 bpm to induce persistent AF. Dogs were used for *in vitro* study after they had been in persistent AF for ≥5 days.

### Atrial myocyte preparation

At the end of the experiments, the dogs were anaesthetized with pentobarbital sodium (30 mg/kg i.v.) and sternotomies were performed. The hearts were quickly removed, and parts of the left atrial wall samples were rapidly frozen in liquid nitrogen and separately stored at −80°C for further analysis. One aliquot of each tissue sample was used to investigate mRNA expression of target genes, whereas the other part was used to determine protein levels. At the same time, their hearts were rinsed in oxygenated Ca^2+^-free Tyrode's solution (mmol/l): NaCl 137; KCl 5.4; MgCl_2_ 1.0; NaH_2_PO_4_ 0.33; HEPES 10; and Glucose 10 (pH 7.4, NaOH). The aortae were cannulated and the hearts were retrogradely perfused on a Langendorff apparatus at 37°C. A perfusion of Ca^2+^-free Tyrode's solution for 5 min was followed by Ca^2+^-free Tyrode's solution containing 0.03% collagenase-II (Worthington Biochemical, Lakewood, CO, USA) and 1% bovine serum albumin (BSA) for 35 min. The left atrium (LA) were dissected, minced and gently triturated with a pipette in a Ca^2+^ Tyrode's solution containing 1% BSA at 37°C for 10 min. The cells were filtered through a 200 μm nylon mesh, and resuspended in the Tyrode's solution in which the Ca^2+^ concentration was gradually increased to 1.0 mmol/l. Only cells with rod-shaped morphology and clear cross-striation were used for experiments.

### Cellular electrophysiological studies

Cells of the LA in a 1 ml bath were continuously superfused (2–3 ml/min.) with normal Tyrode's solution containing (mmol/l): NaCl 137, KCl 5.4, MgCl_2_ 1.0, CaCl_2_ 1.8, NaH_2_PO_4_ 0.33, HEPES 10 and glucose 10 (pH was adjusted with NaOH to 7.4). The solution was bubbled with 100% O_2_. Membrane currents and AP were recorded using whole-cell patch-clamp techniques with an EPC 10 Double amplifier (HEKA, Lambrecht, Pfalz, Germany) and Patchmaster software. Patch pipette resistances ranged from 2.0 to 3.0 MΩ, when filled with an internal solution. The AP was recorded in current-clamp mode. The solution for AP recording (mmol/l) was NaCl 137, KCl 5.4, MgCl_2_ 1.0, CaCl_2_ 1.8, HEPES 10 and Glucose 20 (pH was adjusted with KOH to 7.4). The electrode internal solution for AP recording was KCl 140, MgCl_2_ 2.0, egtazic acid 2.0, HEPES 5.0, EGTA 5 and Na_2_ ATP 4.0 (pH was adjusted with KOH to 7.4). Calcium currents were recorded in the voltage-clamp mode. The external solution for I_Ca-L_ recording contained (mmol): Choline-Cl 137, CaCl_2_ 2.0, MgCl_2_ 1.0, HEPES 5, Glucose 10, CsCl 4.6, TEA-Cl 10, and 4-AP 5 (pH 7.30, CsOH).The internal solution for ICa-L recording contained (mmol): CsCl 120, MgCl_2_ 1.0, MgATP 5.0, BAPTA 10, HEPES 10 and TEA-Cl 10 (pH 7.30, CsOH). In this study, we started data acquisition 10 min. after membrane rupture. I_Ca-L_ magnitudes were normalized by each cellular membrane capacitance (pF) and expressed as current density (pA/pF). Recordings were filtered at low pass (2 Hz) and high pass (30 Hz). Activation voltage dependence was assessed from depolarization-induced currents, with driving force corrected by dividing TP-Erev, where Erev is the voltage axis intercept of the ascending limb of the current-voltage relation. Inactivation was assessed with 1-sec. prepulses from −60, −50, −40, −35, −30, −25, −20, −15, −10, 0, 10, 20, 30 and 40 mV, followed by 240-ms test pulses to +10 mV. The Boltzmann equation was used to fit data. I_Ca-L_ recovery was studied with paired 240-ms pulses to 10 mV (0.1 Hz) delivered at a progressively increasing interpulse interval (P-P) ranging from 3, 5, 8, 10, 20, 40, 60, 80, 160, 300, 500 to 1000 ms.

### Detection of gene expression

Total RNA was extracted from samples of the LA free wall using TRIZOL(Invitrogen Life Technologies, Carlsbad, CA, USA). Expression levels of target genes were measured by real-time quantitative reverse transcription-polymerase chain reaction (qRT-PCR) using SybrGreen qPCR Master Mix (Bio-Rad, Hercules, CA, USA) in a 20 μl reaction volume containing 50 ng of cDNA. All reactions were performed in triplicate and included negative controls. PCR were carried out using an ABI Prism 7500 Sequence Detection System (Applied Biosystems, Carlsbad, CA, USA). Cycling conditions were as follows: 2 min. at 50°C, 10 min. at 95°C, and 40 cycles of 15 sec. at 95°C and 1 min. at 60°C. Relative quantification of mRNA levels was obtained by the 7500 system software using comparative methods. Fluorescence signals were normalized to the housekeeping gene β-actin. The comparative threshold-cycle (CT) relative quantification method was used (ΔΔCT). For each sample, each gene was quantified in duplicate in three separate experiments. The values were averaged and then used to calculate 2^−ΔΔCT^, which corresponds to the expression relative to β-actin. The expected size amplicons were confirmed by gel electrophoreses. The sequences of the genes studied were obtained from GenBank, and the primers were designed using Primer 5.0 software (Applied Biosystems). The amplicon size of the primer sequence and annealing temperature of the genes are shown in Table 1.

**Table 1:** Primer sequence and amplicon size of genes

Gene	Primer sequence	Amplicon size (bp)	Annealing temperature (°C)
β-actin	F: 5′-AAGGACCTGTATGCCAACACA-3′	152	57
	R: 5′-ATCCACACAGAATACTTGCGTT-3′		
LVDCCa1c	F: 5′-GACGCTATGGGCTATGAGTTAC-3′	199	58
	R: 5′-AGTCCAGGTAGCCCTTTAGGT-3′		
Ca^2+^-ATPase	F: 5′-TGGATTACAATGAGGCGAAG-3′	112	56.5
	R: 5′-AGACCCTTCAATTCGGTATCA		
PLN	F: 5′-CACAAGAGCCAAGGCTACCT-3′	135	58
	R: 5′-CAGGAAAGCAGGAAGTCTCAA-3″		
R_Y_R_2_	F: 5′-ATTGAGAAACGATTTGCCTACA-3′	116	57.5
	R: 5′-GGGAAATGTTCTCCTTTGCTT-3′		
IP_3_R_1_	F: 5′-ACGCTATGGGCTCGGTAGTTA-3′	140	57
	R: 5′-ACAGAATACTTGCTTCTCCTT-3′		

### Assessment of protein expression

Membrane protein was extracted from tissue samples of LA with 5 mmol/l Tris-HCl (pH 7.4), 2 mmol/l ethylenediamine tetraacetic acid (EDTA), 5 μg/ml leupeptin, 10 μg/ml benzamidine and 5 μg/ml soybean trypsin inhibitor, followed by tissue homogenization. All procedures were performed at 4°C. Equal amounts (100 μg/sample) of LA membrane proteins were separated on 8% sodium dodecyl sulphate-polyacrylamide gel electrophoresis (SDS-PAGE) gels and transferred to polyvinylidine difluoride membranes. Membranes were blocked in 5% non-fat dry milk in TTBS (Tris-HCl 50-mmol/l, NaCl 500-mmol/l; pH 7.5, 0.05% Tween-20) for 2 hrs at room temperature and then incubated with primary antibody (1:500 dilution) in 5% nonfat dry milk in TTBS for 4 hrs at room temperature. Membranes were then incubated with the following antibodies: rabbit polyclonal anti-Cav1.2 (LVDCCa1c; Santa Cruz Biotechnology Inc, Santa Cruz, CA, USA), rabbit polyclonal anti-IP_3_R_1_ (Santa Cruz Biotechnology), rabbit polyclonal anti-Ca^2+^-ATPase (Santa Cruz Biotechnology), rabbit polyclonal anti-PLN (Abcam, Cambridge, MA, USA) and mouse monoclonal anti-R_Y_R_2_ (Abcam). Membranes were washed three times in TTBS, reblocked in 5% non-fat dry milk in TTBS (15 min.) and then incubated with horseradish peroxidase-conjugated goat anti-rabbit or goat antimouse IgG secondary antibody (1:5000) in 5% non-fat dry milk in TTBS (40 min.). Immunoreactive bands were detected by Immun-Star horseradish peroxidase (HRP) substrate (Bio-Rad) and quantified by densitometry analysis using an Image Quant 350 imager and Image Quant TL-1 software (GE Healthcare, Fairfield, CT, USA). Anti-β-actin antibody (Santa Cruz Biotechnology) was used to control for equal protein loading and to normalize ion channel protein band intensity. All Western blot target bands were quantitatively expressed by normalization to the control band on the same lane. Western blot band intensities were expressed as optical density (OD) units corresponding to densitometric band intensity following background subtraction divided by β-actin signal intensity for the same sample.

### Statistical analysis

Action potential characteristics measured were maximum diastolic potential (MDP), amplitude of phase 0 (APA), plateau potential and APD to 90% repolarization (APD_90_). Quantitative data were presented as mean ± SD. Comparisons between the quantitative data were made using anova. *P* < 0.05 was considered statistically significant. Software SPSS 15.0 was used for statistical analysis (SPSS Inc., Chicago, IL, USA).

## Results

### ECG data

The ECGs of the old dogs manifested longer P-wave durations and P-wave dispersion than adults (66.1 ± 6.4 ms *versus* 75.9 ± 5.3 ms; 19.1 ± 4.1 ms *versus* 26.7 ± 3.1 ms, *n* = 7, all *P* < 0.05, respectively). Other variables did not differ. There was no difference between the two groups in time to onset of persistent AF, the adult dogs developed persistent AF after 40 ± 5 days and old dogs after 52 ± 7 days of atrial pacing (*P* > 0.05).

### Changes in AP characteristics

**Fig. 1:**
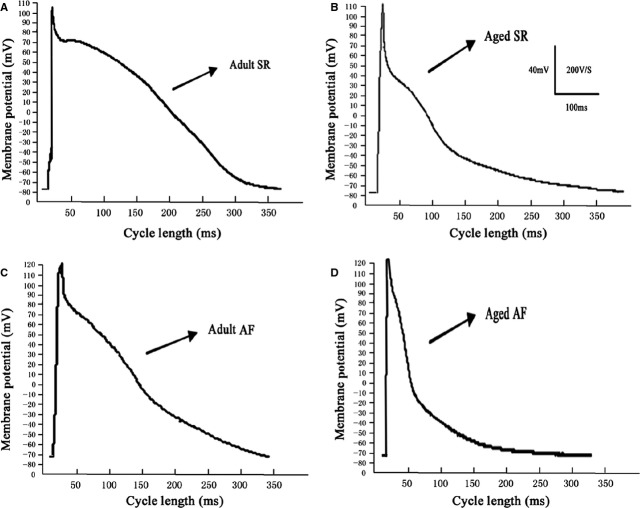
AP recording from LA cardiomyocytes of adult SR canines (**A**), aged SR canines (**B**), adult AF canines (**C**), and aged AF canines (**D**). AP: action potential; LA: left atira; SR: sinus rhythm; AF: atrial fibrillation. (SR adults: 24 cells of seven dogs, SR aged: 30 cells of seven dogs, AF adults: 28 cells of seven dogs, AF aged: 26 cells of seven dogs).

Representative recordings and summary data for major AP parameters of adult and aged dogs in SR and those with AF are shown in Table 2 and Figure 1. Cardiomyocytes from aged atria were longer APD_90_, AP plateau potential was significantly lower in comparison to adults, while there were no significant differences in MDP and APA. APD_90_ was shortened with AF in both adult and aged groups with more shortening in the latter resulting in no difference between APD_90_ in both AF groups. AF led to a higher MDP, a significant increase in APA and a lower of AP plateau potential at both ages. AF was associated with a significant depolarization of the cellular membrane in both adult and aged LA. The extent of depolarization was the same in both AF groups leaving adult cardiomyocytes more depolarized than aged cardiomyocytes.

**Table 2:** AP characteristics recorded from adult and old atria in SR and AF at a cycle length of 2000 ms

Group	*n*	MDP (mv)	APA (mv)	Plateau (mv)	APD_90_ (ms)
SR adult	24	−78.8 ± 0.8	109.8 ± 1.4	−4.0 ± 0.7	320.0 ± 7.9
SR aged	30	−79.2 ± 1.4	110.5 ± 4.9	−7.5 ± 1.7*	340.5 ± 10.1*
AF adult	28	−71.8 ± 0.9*	121.8 ± 1.1*	−6.4 ± 1.1*	297.0 ± 5.6*
AF aged	26	−72.2 ± 1.2†	122.5 ± 2.9†	−9.8 ± 1.1†	300.5 ± 7.1†

* *P* < 0.05, compared with the adult SR group.

† *P* < 0.05, compared with the aged SR group.

Data are presented as mean ± SD.

AP: action potential; MDP: maximum diastolic potential; APA: action potential amplitude; APD_90_: action potential duration to 90% repolarization; SR: sinus rhythm; AF: atrial fibrillation; n: the number of cells of each group (SR adult: 24 cells of seven dogs, SR aged: 30 cells of seven dogs, AF adult: 28 cells of seven dogs, AF aged: 26 cells of seven dogs).

### Changes in I_Ca-L_ characteristics

**Fig. 2:**
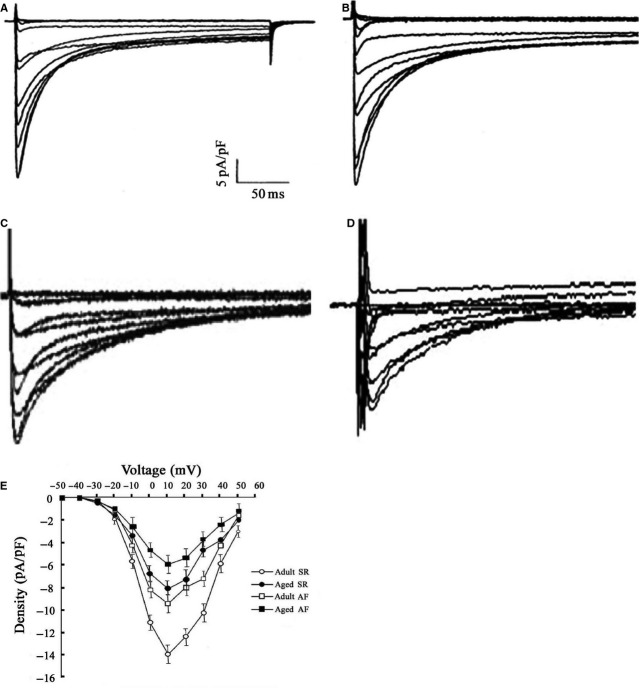
I_Ca-L_ tracings between adults and aged LA in SR and AF, holding voltage of −70 mV to various test voltages, adult SR canines (**A**), aged SR canines (**B**), adult AF canines (**C**), and aged AF canines (**D**); LA: left atira; SR: sinus rhythm; AF: atrial fibrillation. (**E**) Average peak I_Ca-L_ density in adult and aged cells. All data were collected at the same time after establishing whole cell configuration (adults, 17 ± 0.8 min.; aged, 18 ± 1.1 min.). (SR adult: 14 cells of seven dogs, SR aged: 16 cells of seven dogs, AF adult: 15 cells of seven dogs, AF aged: 19 cells of seven dogs).

Typical recordings of I_Ca-L_ and comparative major I_Ca-L_ parameters of adults and aged dogs in SR and those with AF are shown in Table 3 and Figure 2. Aged LA cardiomyocytes demonstrated lower peak I_Ca-L_ densities than adult LA cells. This decrease tendency was the same in the both adults and aged groups in AF, while the latter appeared to be lower. Activation voltage dependence had no significant difference in half-activation voltage and slope factor of each group; also, inactivation had no significant difference in half-inactivation voltage and slope factor of each group. Otherwise, this current reduction during ageing and in AF was unaccompanied by a significant change in its recovery time from inactivation.

**Table 3:** Electrophysiological characteristics of I_Ca-L_ between adult and aged LA in SR and AF

			Steady-state activation		Steady-state inactivation		
					
Group	*n*	I_Ca-L_ density (pA/pF)	V_0.5_ (mV)	k (mV)	V_0.5_ (mV)	k (mV)	Monoexponential recovery time constants (ms)
SR adult	14	−14.1 ± 0.8	−7.1 ± 1.5	5.7 ± 0.4	−23.1 ± 2.1	6.2 ± 0.3	51.9 ± 3.3
SR aged	16	−8.1 ± 0.5*	−6.7 ± 2.8	5.5 ± 0.5	−22.9 ± 3.3	6.4 ± 0.5	53.1 ± 3.1
AF adult	15	−9.4 ± 0.7*	−6.9 ± 1.2	5.1 ± 0.3	−22.1 ± 1.9	6.2 ± 0.3	51.2 ± 2.3
AF aged	19	−5.9 ± 0.3†	−6.8 ± 2.1	5.9 ± 0.3	−21.9 ± 2.3	6.8 ± 0.6	52.1 ± 5.1

* *P* < 0.05, compared with the adult SR group.

† *P* < 0.05, compared with the aged SR group.

Data are presented as mean ± SD.

V_0.5_ and *k* are average values of voltage at half maximal availability and slope factor as determined using a Boltzmann equation. I_Ca-L_ current densities at maximal voltage (−90 mV) are shown.

LA: left atira; SR: sinus rhythm; AF: atrial fibrillation; n: the number of cells of each group (SR adult: 14 cells of seven dogs, SR aged: 16 cells of seven dogs, AF adult: 15 cells of seven dogs, AF aged: 19 cells of seven dogs).

### Left atrial mRNA and protein expressions of proteins influencing calcium homeostasis

**Fig. 3:**
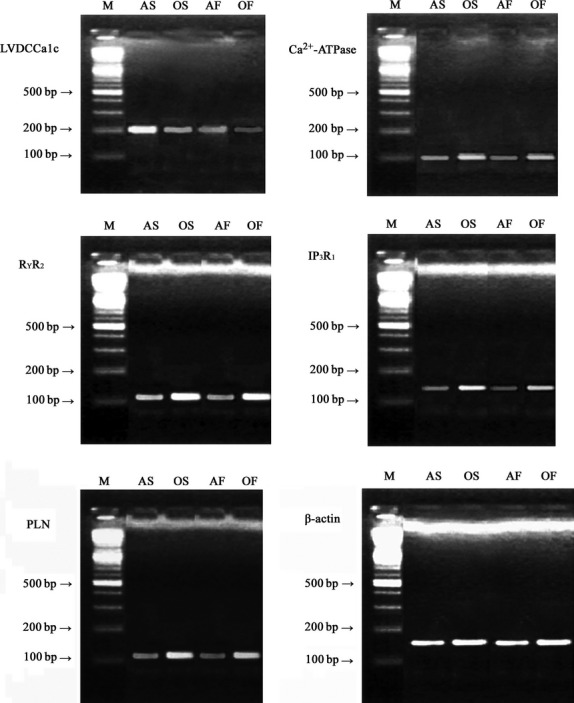
Representative gels of Ca^2+^ handling protein (LVDCCa1c, L-type calcium channel a1c; Ca^2+^-ATPase, calcium adenosine triphosphatase; R_Y_R_2_, ryanodine receptor type-2; IP_3_R_1_, inositol triphosphate receptor type-1; PLN, phospholamban) and β-actin in the LA myocardium between adult and aged groups in SR and AF. LA: left atira; SR: sinus rhythm; AF: atrial fibrillation; M: marker; AS: Adult SR group; OS: Old SR group; AF: Adult AF group; OF: Old AF group.

**Fig. 4:**
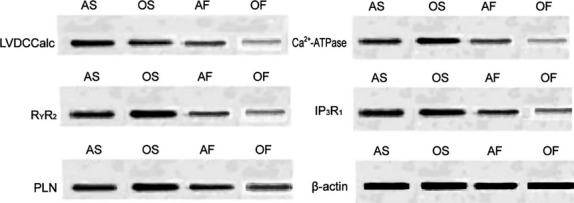
Representative immunoblots (Western blots) showing Ca^2+^ handling protein expression (LVDCCa1c, L-type calcium channel a1c; Ca^2+^-ATPase, calcium adenosine triphosphatase; R_Y_R_2_, ryanodine receptor type-2; IP_3_R_1_, inositol triphosphate receptor type-1; PLN, phospholamban) and β-actin in the LA myocardium between adult and aged groups in SR and AF. LA: left atira; SR: sinus rhythm; AF: atrial fibrillation; AS: Adult SR group; OS: Old SR group; AF: Adult AF group; OF: Old AF group.

As shown in Tables 4 and 5 and Figures 3 and 4, compared to the adult group, mRNA and protein expressions of LVDCC_a1c_ were significantly decreased, mRNA and protein expressions of Ca^2+^-ATPase were significantly increased in the aged group (all *P* < 0.05). Moreover, mRNA and protein expressions of R_Y_R_2_, IP_3_R_1_ and PLN showed up-regulation tendency in the aged group, but were not significantly greater in the two groups (all *P* > 0.05).

**Table 4:** The expressions of mRNA in the left atrial myocardium between adult and aged LA in SR and AF

Group	*n*	LVDCCa1c	Ca^2+^-ATPase	R_Y_R_2_	IP_3_R_1_	PLN
SR adult	7	2.38 ± 1.03	1.14 ± 0.83	2.49 ± 1.02	2.68 ± 0.97	1.72 ± 0.71
SR aged	7	1.17 ± 0.75*	2.32 ± 0.75*	3.63 ± 0.89	3.12 ± 1.21	1.97 ± 0.84
AF adult	7	0.27 ± 0.25*	0.30 ± 0.12*	0.52 ± 0.21*	0.85 ± 0.21*	1.28 ± 0.94
AF aged	7	0.10 ± 0.07†	0.17 ± 0.07†	0.26 ± 0.09†	0.67 ± 0.19†	1.46 ± 0.52

* *P* < 0.05, compared with the adult SR group.

† *P* < 0.05, compared with the aged SR group.

Data are presented as mean ± SD.

LVDCCa1c: L-type calcium channel a1c; Ca^2+^-ATPase: calcium adenosine triphosphatase; R_Y_R_2_: ryanodine receptor type-2; IP_3_R_1_: inositol triphosphate receptor type-1; PLN: phospholamban; LA: left atira; SR: sinus rhythm; AF: atrial fibrillation.

**Table 5:** The expressions of protein in the left atrial myocardium between adult and aged LA in SR and AF

Group	*n*	LVDCCa1c	Ca^2+^-ATPase	R_Y_R_2_	IP_3_R_1_	PLN
SR adult	7	0.28 ± 0.11	0.36 ± 0.08	0.23 ± 0.04	0.28 ± 0.07	0.32 ± 0.09
SR aged	7	0.13 ± 0.10*	0.48 ± 0.13*	0.26 ± 0.05	0.35 ± 0.06	0.36 ± 0.08
AF adult	7	0.13 ± 0.01*	0.25 ± 0.07*	0.17 ± 0.04*	0.17 ± 0.01*	0.31 ± 0.04
AF aged	7	0.07 ± 0.05†	0.13 ± 0.03†	0.10 ± 0.02†	0.15 ± 0.04†	0.31 ± 0.05

* *P* < 0.05, compared with the adult SR group.

† *P* < 0.05, compared with the aged SR group.

Data are presented as mean ± SD.

LVDCCa1c: L-type calcium channel a1c; Ca^2+^-ATPase: calcium adenosine triphosphatase; R_Y_R_2_: ryanodine receptor type-2; IP_3_R_1_: inositol triphosphate receptor type-1; PLN: phospholamban; LA: left atira; SR: sinus rhythm; AF: atrial fibrillation.

Compared to control groups, mRNA and protein expressions of LVDCC_a1c_ were significantly decreased; moreover, mRNA and protein expressions of Ca^2+^-ATPase, R_Y_R_2_ and IP_3_R_1_ were significantly decreased in both adult and aged groups in AF (all *P* < 0.05), while mRNA and protein expressions of PLN showed a down-regulation tendency, but had no significant difference in two groups (*P* > 0.05).

## Discussion

### Ageing-associated changes of LA electrophysiology in SR

Interestingly, our study showed that the most remarkable alteration with ageing was a significant lowering of the AP plateau potential and APD_90_ was prolonged in aged LA cardiomyocytes. Moreover, P wave duration and dispersion were significantly longer in the aged canines. The result might be a reflection of ageing-associated degree of slower conduction of atria. Previous studies have reported that I_Ca-L_ is reduced in aged canine right atria cardiomyocytes compared to adults [3], but there are no published data on the effects of age on LA cardiomyocytes I_Ca-L_. Our study demonstrated that there was a significant reduction in peak I_Ca-L_ in aged canine LA cardiomyocytes, while decreased LVDCC_a1c_ protein levels might be the major reason of the reduced I_Ca-L_. However, the current reduction in aged atrial cardiomyocytes was unaccompanied by a significant change in calcium channel availability or recovery from inactivation. The currents which determine the plateau level of AP in atria are I_Kur_, I_to_ and I_Ca-L_ [15, 16]. Therefore, a decrease in depolarizing current I_Ca-L_ or an increase in repolarizing currents (I_Kur_ and/or I_to_) may lead to the lower plateau of AP. So the result suggested that the decrease in I_Ca-L_ may be a major mechanism for the low plateau potential in aged canine LA cardiomyocytes. The longer APD_90_ in old atria suggested some ageing-induced changes of delayed rectifier potassium (I_K_) or might be simply a consequence of the low plateau potential in aged dogs. Previous study revealed that negative plateau potentials had a lower driving force for conduction of early premature beats [17, 18].Therefore, our results implied that the change in AP in old atria would lead to a decreased conduction of premature beats. Slow conduction of early premature impulses might well further facilitate the onset of AF.

### Impact of AF on electrophysiology of adult and old LA

Many experimental studies have demonstrated that AF remodels atrial electrophysiology to facilitate its own recurrence [19, 20]. The major electrophysiological characteristics of electrical remodelling are reduction in the atrial refractory period and loss of APD adaptation to rate [21, 22]. To date, studies have been performed in normal adult animals. AF-induced electrophysiological remodelling in adults results from rapid atrial activation, and rapid atrial pacing produces similar AP changes. Yet, the mechanism for AP changes seen with this remodelling may differ, as shown in our study. In our study, we found that there was reduced I_Ca-L_ in persistent AF cardiomyocytes *versus* controls. The change was interpreted as resulting from reduced LVDCC_a1c_ protein levels. It appears reasonable to propose that calcium ion channel remodelling is the basis of the atrial electrical remodelling of AF. On the basis of the present results, it was found that AF led to a remarkable shortening of APD_90_, a higher MDP, a significant increase in APA and a significant decrease in AP plateau potential at both ages. AF-induced decreases in APD_90_ might be explained by the reduction in I_Ca-L_, AF was associated with a significant depolarization of the cellular membrane in both adults and aged LA. Such membrane depolarization in AF may be a consequence of decreased basal I_Ca-L_.

### Ageing-associated changes of molecular biology of LA in SR and in AF

In this study, it is noteworthy that LVDCC_a1c_ expression levels were significantly downregulated, however, intracellular Ca^2+^ handling proteins generally showed an up-regulation tendency during ageing; moreover, the expression levels of LVDCC_a1c_ and intracellular Ca^2+^ handling proteins except PLN were are highly downregulated in the adult and aged groups in AF, more specifically in the latter with AF. In the intact heart, electrical and mechanical alternans are most frequently observed during acute myocardia in AF, a condition that is likely to affect glycolytic metabolism through restricted substrate availability [23–27]. As a result, the phosphorylation reactions are slowed and the availability of active RyR_2_ channels on a beat-to-beat basis is reduced [28, 29]. Therefore, the hypothesis of attenuated energy production based on glycolytically derived ATP remains an intriguing possibility to explain the increased susceptibility of aged atria to AF. It is known that sarcoplasmic reticulum Ca^2+^ circulation and the activity of Ca^2+^ handling proteins are regulated by phosphorylation processes [26, 28–30]. On the basis of our results, it appears reasonable to propose that due to ageing, especially in the occurrence of AF, the rate of phosphorylation of Ca^2+^ handling proteins is slowed and the equilibrium between phosphorylated and non-phosphorylated channels is shifted towards the non-phosphorylated state.

In conclusion, these age-associated electrophysiological and molecular changes have suggested that ageing reduces the activity of Ca^2+^ handling proteins, abnormal Ca^2+^ handling might be due to impaired phosphorylation-dependent regulation of these multi-step Ca^2+^ handling proteins, and this up-regulation tendency with ageing is probably a physiological adaptation mechanism. AF is also associated with the elaborate adaptive and maladaptive reactions in the electrophysiology, functional ion-current and ion-channel gene and protein expression changes. The exact mechanism remains to be elucidated.

### Study limitations

First, the plateau potential of AP was determined by I_Kur_, I_to_ and I_Ca-L_, but our study did not included I_Kur_ and I_to_. Second, APA is related with the I_Na_, but we did not study the change in I_Na_. Finally, our findings were limited to the studies of cardiomyocytes from LA and cannot necessarily be extended to cells of other regions of the atria. Also, due to that the function of the intracellular Ca^2+^ handling protein was fully studied, it was not repeated in the discussion. Ageing is also characterized by a progressive deterioration in physiological functions and metabolic processes, which may alter the amount and distribution of ion channels. Further the extent to which the altered electrophysiological properties seen with ageing may be arrhythmogenic and increase the likelihood of AF is presently unknown.
